# Is female genital mutilation associated with eclampsia? Evidence from a nationally representative survey data

**DOI:** 10.1186/s12978-020-00918-7

**Published:** 2020-05-20

**Authors:** Saverio Bellizzi, Lale Say, Arash Rashidian, Michel Boulvain, Jasmine Abdulcadir

**Affiliations:** 1 Partnership for Maternal, Newborn and Child Health, Geneva, Switzerland; 2 Department of Reproductive Health and Research, World Health Organization, Geneva, Switzerland; 3 Department of Information, Evidence and Research, World Health Organization Regional Office for the Eastern Mediterranean, Cairo, Egypt; 4 Department of woman, child and adolescent. Division of Gynecology, Geneva University Hospitals, Geneva, Switzerland

**Keywords:** Eclampsia, Female genital mutilation, Demographic health survey

## Abstract

**Background:**

Studies have shown the impact of female genital mutilation (FGM), especially infibulation (WHO type III), on reproductive health, and adverse obstetric outcomes like postpartum haemorrhage and obstructed labour. However, whether an association exists with maternal hypertensive complication is not known. The present study sought to investigate the role of the different types of FGM on the occurrence of eclampsia.

**Methods:**

The study used data from the 2006 Demographic and health survey of Mali. The proportion of eclampsia in women with each type of FGM and the unadjusted and adjusted odds ratios (OR) were calculated, using women without FGM as reference group. Unadjusted and adjusted OR were also calculated for women who underwent infibulation compared to the rest of the population under study (women without FGM and women with FGM type I, II, and IV).

**Results:**

In the 3997 women included, the prevalence of infibulation was 10.2% (*n* = 407) while 331 women did not report FGM (8.3%). The proportion of women reporting signs and symptoms suggestive of eclampsia was 5.9% (*n* = 234).

Compared with the absence of female genital mutilation and adjusted for covariates, infibulation was associated with eclampsia (aOR 2.5; 95% CI:1.4–4.6), while the association was not significant in women with other categories of FGM. A similar aOR was found when comparing women with infibulation with the pooled sample of women without FGM and women with the other forms of FGM.

**Conclusion:**

The present study suggests a possible association between infibulation and eclampsia. Future studies could investigate this association in other settings. If these findings are confirmed, the possible biological mechanisms and preventive strategies should be investigated.

## Plain English summary

Female genital mutilation (FGM), which includes any procedure involving the alteration or excision of external female genitalia without medical indication, represents a violation of human rights of women and is a major public health problem in several countries in Africa and Middle East. FGM have multiple adverse consequences, from psychological to reproductive health. In consideration of the association between FGM and conditions like urinary tract infections as well as the association between inflammatory processes and placentation, we explored the effect of female genital mutilation on the occurrence of eclampsia. We used self-reported information on the four different types of FGM and about signs and symptoms compatible to eclampsia for 3997 women, included in the Mali Demographic and Health Survey, who gave birth during the twelve months prior to the interview. FGM with genital area sewn closed (infibulation) was associated with increased risk of eclampsia, even when adjusted for potential confounders. This association was also present when comparing women with infibulation to women without FGM and women with other forms of FGM. Women with other types of FGM did not have a significantly higher risk of developing eclampsia. Such findings need further confirmation and may have important implications on treatment of FGM like the de-infibulation that performed at the beginning of pregnancy may reduce the risk of hypertensive disorders.

## Introduction

Female genital mutilation (FGM), also named female genital cutting, is acknowledged as a violation of human rights of women [1]. The United Nations Sustainable Development Goals called for the elimination of the practice by 2030 [2]. FGM is referred to as any procedure involving the alteration or excision of external female genitalia without medical indication [3], and 3 million women in the world are estimated to be at risk of undergoing this procedure annually [3]. It is a major public health problem in several countries in Africa and Middle East [3, 4], being almost universal in seven African countries (prevalence > 85%) [3].

Studies carried in different settings have clearly showed an adverse effect of FGM on psychological, sexual and reproductive health unfavourable outcomes [5]. This includes post-traumatic stress disorder [6], dyspareunia and genitourinary complications. Adverse obstetric outcomes, such as increased risk for caesarean delivery, episiotomy and postpartum haemorrhage, are also more frequent [7, 8]. Scar tissue, especially in women with FGM type III (infibulation) can result in obstructed labour or obstetric trauma [9].

Pre-eclampsia occurs in around 2–8% of all pregnancies [10] and represents one of the major challenges for researchers in terms of etiology and physiological mechanisms; however, the central role of the placenta in its pathogenesis is undisputed [11]. ‘The two-stage theory’ is widely accepted with regard to mechanisms of preeclampsia [12]. In preeclampsia, the transformation of the maternal uterine spiral arteries into larger diameter vessels with low resistance to blood flow is impaired (abnormal placentation). This is most likely due to immunological and environmental factors. Immunological factors, involve the activation of cells like the cytotoxic Natural Killer cells, which cause an increase in innate immune activation and inflammation [13]. The abnormal placentation leads to the release of ‘placental factors’ in the maternal circulation, producing an imbalance in immune functions that leads to chronic inflammations and generalized endothelial dysfunction [12].

No previous scientific literature has investigated preeclampsia and eclampsia among women with different types of FGM. FGM has been found to be associated with inflammatory and infectious processes like urinary tract infections (unadjusted RR = 3.0) and bacterial vaginosis (adjusted OR = 1.7) [14]. Specifically, infibulation creates a bridge of skin which obscures the opening of the urinary canal, which causes deflection of the normal flow of urine with the area remaining constantly wet and susceptible to bacterial infection [15].

One might hypothesize a possible negative effect of FGM on placentation process, due to inflammation in case of repeated genitourinary infections, especially in the case of FGM type III.

Using data of the Demographic Health Survey (DHS) program, we sought to assess the association between reported female genital mutilation and the occurrence of signs and symptoms suggestive of eclampsia during the last pregnancy.

## Methods

### Population, setting and data

We used data from the Demographic and Health Survey (DHS) international project, which is implemented by Macro International and funded by the United States Agency for International Development [5].

DHS are nationally representative random household surveys including several health indicators, with a particular focus on maternal and child health [16]. All women of reproductive age (15–49 years) are the target population in most DHS surveys. DHS guidelines are designed to maximize safety and disclosure, including interviewing only one woman per household, and maintaining complete privacy during the interview [17].

Because of the high prevalence of FGM and the high incidence of pre-eclampsia/eclampsia in the Sub-Saharan countries, we focused on these countries [3]. The study inclusion criteria were that the country-specific DHS data set included both the modules for female circumcision and obstetric complications. Our analysis had to be limited to the Mali DHS (2006), which met these inclusion criteria.

The Mali DHS used a two-stage clustered sampling based on national census data and provides data on a nationally representative sample [18]. The survey was administered from May through December 2006 to 12,998 out of a total 13,160 randomly selected households (98.8% response rate) [5]. Furthermore, 14,583 out of a total 15,102 women aged 15–49 participated in the survey, yielding a 96.6% response rate.

For our analysis we only considered the latest pregnancy that occurred within the twelve months period prior to the survey, thus excluding 10,388 women. After excluding records with missing data on FGM and on the outcome (e.g. convulsions) and on important covariates (e.g. twin pregnancies) [19], the analysis included 3997 individuals (Fig. 1).

### Main outcome, exposure and other variables

We used women self-reported occurrence of convulsions not caused by fever as a proxy for the outcome (eclampsia) and women self-reported FGM as the exposure. The index pregnancy corresponds with the closest pregnancy to the DHS interview in case two pregnancies occurred in the 12 months period.

During the interview, women were first asked whether they know of FGM, with those who are familiar questioned on whether they have been cut themselves [20]. Respondents reporting they have been cut are asked whether any flesh was removed from their genitals and, if so, if their genital area was sewn closed. These questions allow to assign these women to the WHO FGM type 1 or 2 (excision of the clitoral hood and/or the visible part of the clitoris and/or the labia), and to the type 3 (infibulation, the narrowing of the vaginal orifice by apposition of the labia, with or without excision of the external part of the clitoris) [21–23]. Similarly, participants reporting no flesh was removed are asked whether their genitals were “nicked” without flesh removal [21], corresponding to the WHO FGM type 4 [24].

We considered the following variables as potential confounders: maternal age categorized into three groups, from age 15 to age 24, from age 25 to age 36, and from age 37 to age 49; place of residence split into urban and rural; a wealth index based on asset-ownership and household characteristics (categorized using the quintiles “poorest”, “poorer”, “middle”, “richer”, and “richest”) was considered as a proxy for socio-economic status [25]. Maternal educational attainment was included after classification in “no education”, “primary”, “secondary”, and “higher” [26]. Because of a strong association with maternal hypertensive complications, access to antenatal care and parity were included in the analyses [27].

### Statistical analysis

We use counts and percentages to describe the occurrence of eclampsia and FGM. We also described the distribution of each covariate by reported FGM. We evaluated the prevalence of FGM across the levels of the covariates in the analysis using cross tabulations and computed the *p*-value using the Chi-squared, Fisher exact and Chi-squared for trend tests. Then, to evaluate the risk of eclampsia in each group of FGM (without flesh removed, with flesh removed and with genital area sewn closed), we computed unadjusted and adjusted odds ratios (OR), with women without FGM as reference. An analysis was also conducted comparing FGM type III (infibulation) with the pooled population of women without FGM and women with FGM type I, II and IV.

We used logistic regression modelling to adjust for maternal age, residence, wealth, maternal education, access to ANC and birth order. We also accounted for within-cluster correlation by taking into consideration the primary sampling units [28].

We used Stata13.1 SE (Stata Corp LP, College Station, Texas, USA) for statistical analysis [29].

## Results

Of the 3997 women in the dataset, the prevalence of reported FGM without flesh removal was 4.2% (*n* = 167), with flesh removal 77.3% (*n* = 3092) and with genital area sewn closed 10.2% (*n* = 407); 331 women did not report having FGM (8.3%). The proportion of women self-reporting convulsions with no fever around childbirth was 5.9% (*n* = 234).

There were no differences in self-reported FGM by categories of age. FGM was slightly more frequent in rural areas. A significant linear trend appeared when assessing wealth and education, with fewer FGM for richest and most educated women (Table 1). No significant difference was detected when exploring access to ANC consultations and successive birth order position.

**Table 1:** Descriptive characteristics of interviewees and association with FGM in Mali, 2006, *n* = 3997

Variable		Reporting FGM		
	Total	n	%	P-value
Age in years				
15–24	1418	1302	91.8	0.9
25–36	1682	1540	91.6	
37–49	897	824	91.9	
Residence				
Urban	2112	1919	90.9	0.04
Rural	1885	1747	92.7	
Wealth quintile				
Poorest	501	483	96.4	< 0.001
Poorer	569	540	94.9	
Middle	613	554	90.4	
Richer	930	838	90.1	
Richest	1384	1251	90.1	
Education level				
None	2878	2677	93.0	< 0.001
Primary	648	577	89.0	
Secondary	442	388	87.8	
Higher	29	24	82.8	
ANC				
None	257	241	93.8	0.2
At least one	3740	3425	91.6	
Parity				
First birth	380	349	91.8	0.2
Second or more	3617	3317	90.3	

No significant association was detected when assessing the relationship between eclampsia and FGM with or without flesh removed. FGM with genital area sewn closed (infibulation) was associated with more than twofold increased odds of eclampsia, even when adjusted for potential confounders (Table 2). This association was also present (aOR 2.4; 95% CI 1.7–3.4) when comparing women with infibulation to women without FGM and women with other forms of FGM.

**Table 2:** Association between exposure to FGM and signs and symptoms suggestive of eclampsia occurring in a pregnancy in the previous twelve months, Mali 2006, *n* = 3658

	Eclampsia					
	Total	Yes, n (%)	cOR	95% CI	aOR	95% CI
**No FGM**	331	16 (4.8)	1.0	–	1.0	–
**Just nicked (type I)**	167	4 (2.4)	0.5	0.2–1.5	0.6	0.1–1.6
**Flesh removed (type II)**	3092	166 (5.4)	1.1	0.7–1.9	1.1	0.6–1.9
**Genital area sewn closed (type III, infibulation)**	407	48 (11.8)	2.8	1.5–5.0	2.5	1.4–4.6

cOR: Crude Odds Ratio; aOR: Adjusted Odds Ratio for maternal age, residence, wealth, maternal education, access to ANC, and parity

## Discussion

### Principal findings

Our study shows that women with FGM type III (infibulation) were at higher risk of eclampsia, when compared to women with no FGM. Women with other types of FGM did not have a significantly higher risk of developing eclampsia. Obstetric complications such as prolonged labour, perineal trauma and postpartum haemorrhage in women with FGM type III, are well described [8], but there were no previous study reported the association with maternal hypertensive disorders.

### Clinical implications

A biological hypothesis for this association may be found in the link between infection and eclampsia. Infibulated women are at risk for chronic/recurrent genitourinary infections and Pelvic Inflammatory Disease because of the covering (obstruction) of the vaginal orifice and urethral meatus, which often leads to urinary retention [15]. A recent meta-analysis including 19 studies has shown that urinary tract infection during pregnancy represents a significant risk factor for pre-eclampsia (OR 1.31; 95% CI: 1.22–1.40) [30]. Infections may alter placentation, through the activation of systemic inflammatory response and endothelial injury, causing placental hypoxia, ultimately leading to preeclampsia and eclampsia [30].

Infibulated women can become pregnant without having engaged in penetrative sex [31], and the reduced exposure to seminal fluid via vaginal route might be another possible explanation for higher incidence of eclampsia among women with FGM type III [32]; a growing body of literature in fact suggests that exposure to paternal antigens in seminal fluid via the vaginal mucosa may induce maternal tolerance to the allogeneic fetus, facilitating successful implantation and protecting from preeclampsia caused by immune maladaptation [33].

### Strengths and limitations

DHS are often the only source of maternal health data available in low- and middle-income countries and are considered high-quality surveys due to the use of standardized procedures and questionnaires [34]. However, the validity of self-reported data on FGM and obstetrical complications cannot be guaranteed. This issue has been recognized by other scientists using DHS data for secondary analysis on FGM and maternal complications around birth [35–37]. Despite the fact that accuracy of obstetrical complications is generally low, sensitivity, specificity and likelihood for reported convulsions have been found 96.4 (95% CI 79.8–99.8), 87.5 (95% CI 84.2–90.3) and 7.7 (95% CI 6.4–9.9), respectively [38]. Maternal recall is also influenced by the communication between health provider and patient, including the capacity of health provider to diagnose severe complications accurately [39]. A possible limitation of our study is that the frequency of self-reported FGM and signs/symptoms suggestive of eclampsia in this study may be an underestimation, but this will result in a conservative estimation of the magnitude of the association between FGM and eclampsia, assuming non-differential misclassification. The highly sensitive nature of FGM may also influence reporting of data based on women’s self-reports to trained interviewers, as there is the potential of social desirability bias [5]. However, we believe that bias in reporting will be the same in women who had eclampsia and women who did not.

We were not able to take into consideration the distance of women from a health facility, which might be a confounder in the association under study. We considered the place of residence, dichotomized in urban and rural, as a surrogate to adjust for access to health care.

Additional limitations can be attributed to the fact that data analyzed is more than 10 years old and that no differential diagnosis with other potential causes of convulsions in absence of fever in pregnancy was possible.

## Conclusions

Further and more rigorous studies should be conducted to confirm the association between infibulation and eclampsia and understand the pathophysiological mechanisms. Objective measurements and recognition of clinical signs by health professionals in health-facility based assessments may represent valid options. The confirmation of this association might have an impact on maternal as well as neonatal morbidity and mortality. Eclampsia is an important risk factor for maternal death around the time of birth, especially in low-resourced settings like Sub-Saharan Africa where access to antenatal care and emergency obstetric care is far below the required standards. Additionally, eclampsia is an important determinant of preterm birth, which now account as primary cause of under 5 mortality. If recurrent genital and urinary infections in FGM type III are associated with impaired placentation, de-infibulation before or at the beginning of pregnancy and treatment of genitourinary infection may reduce the risk of hypertensive disorders.

## Data Availability

The dataset is publicly available through formal request mechanisms from ORC Macro (Calverton, MD, USA).

## Abbreviations

ANC: Antenatal care
DHS: Demographic Health Survey
FGM: Female Genital Mutilation
OR: Odds Ratio
aOR: adjusted Odds Ratio
WHO: World Health Organization
